# Vedolizumab for Crohn's disease: Real‐world efficacy through a meta‐analytical lens

**DOI:** 10.1002/ueg2.12690

**Published:** 2024-11-07

**Authors:** Maria Manuela Estevinho, Virginia Solitano

**Affiliations:** ^1^ Department of Gastroenterology Unidade Local de Saúde Gaia Espinho (ULSGE) Vila Nova de Gaia Portugal; ^2^ Department of Biomedicine Unit of Pharmacology and Therapeutics Faculty of Medicine University of Porto Porto Portugal; ^3^ Division of Gastroenterology Department of Medicine Western University Schulich School of Medicine London Ontario Canada; ^4^ Division of Gastroenterology and Gastrointestinal Endoscopy IRCCS Ospedale San Raffaele Università Vita‐Salute San Raffaele Milan Italy

**Keywords:** Crohn's disease, effectiveness, real‐world evidence, targeted therapies, vedolizumab

Over the past 2 decades, Crohn's disease (CD) treatment options have significantly expanded. This began with infliximab's approval in 1998, followed by adalimumab in 2007. For nearly 2 decades no new therapy classes emerged, until the approval of vedolizumab in 2014 and ustekinumab in 2016. The availability of affordable biosimilars for infliximab and adalimumab has reinforced their role as first‐line treatments.^1^ However, up to 30% of patients do not initially respond, and up to 40% may lose effectiveness or develop side effects, necessitating alternative therapies.^2^, ^3^ Conflicting international guidelines on treatment sequencing stem from difficulties in comparing clinical trials with varying designs and the relative absence of head‐to‐head studies. On the other hand, network meta‐analyses rank therapies mostly by clinical remission, but endoscopic remission or mucosal healing are more predictive of long‐term outcomes. Thus, real‐world evidence is crucial to refining therapeutic strategies and algorithms.^4^

To analyze the effectiveness of vedolizumab treatment for CD, Armuzzi et al.^5^ performed a systematic review and meta‐analysis of 73 real‐world studies (comprising 29,894 Crohn's disease patients). In this study, vedolizumab persistence rates were 65.3% and 54.8% at 1 year and 2 years, respectively. Notably, biologic‐naive patients exhibited higher persistence compared to biologic‐experienced patients at both 1 year (82% vs. 63%) and 2 years (71% vs. 41%). Comparative studies also revealed superior persistence with vedolizumab over anti‐TNFα therapies at 1 year (85% vs. 75%) and 2 years (71% vs. 65%). Biologic‐naive patients treated with vedolizumab had higher rates of mucosal healing (53% vs. 36%), clinical remission (66% vs. 35%), and corticosteroid‐free remission (54% vs. 33%) at 1 year. Likewise, in another recently published metanalysis, that analyzed all approved advanced therapies, vedolizumab as first‐line therapy had lower rates of treatment discontinuation or failure after 3 years of follow‐up compared to both infliximab and adalimumab.^1^ These results suggest that reserving vedolizumab for CD patients naïve to anti‐TNF therapy is the best option for maximizing its benefit. In addition, findings from other recent metanalysis^1^ suggest that prior vedolizumab treatment does not significantly affect the effectiveness of anti‐TNF therapy, reinforcing the advantage of using vedolizumab as a first‐line biologic option.

The strength of this work lies in its rigorous methodology, which utilized a comprehensive search strategy to include non‐interventional studies with sample sizes of at least 50 patients. This approach offers valuable insights into real‐world treatment effectiveness and persistence in CD, enhancing the reliability and applicability of the findings. However, this meta‐analysis is not without limitations. First, studies published after the first half of 2022 were not included, yet the authors demonstrated in the discussion that their results remain consistent with more recent findings. Second, significant between‐study heterogeneity was observed in all treatment persistence analyses, which was not further investigated. This could be attributed to differences within the studies, such as variations in age, mean disease duration (ranging between 3 and 19 years), baseline disease activity, and dose‐escalation patterns. These factors, particularly disease duration, are highly relevant. Although no specific cutoff has been established, it would have been valuable to explore whether patients with earlier‐stage disease exhibit higher drug persistence. Regarding dose escalation, while evidence supports this practice,^6^ it is not explicitly included in the label, leading to variability in dosing adjustments across sites. Additionally, unmeasured factors (e.g., patient preference, appearance of extraintestinal manifestations, reimbursement issues) may have influenced treatment persistence rates. Given the limited data and the lack of detailed reporting to enable informative and reliable subgroup analyses, conducting individual‐level meta‐analyses could be a valuable approach to gaining deeper insights. Finally, in this metanalysis, all anti‐TNF drugs (infliximab, adalimumab, golimumab, certolizumab) were grouped together, potentially masking differences in their effectiveness and favoring vedolizumab in the comparisons.

An integrated synthesis of risk and benefit from various evidence sources, including head‐to‐head trials and real‐world data, while considering patients' values and preferences, can guide the optimal positioning of therapies to enhance patient outcomes. Any treatment algorithm proposed for individuals with CD should incorporate data on the comparative effectiveness and safety of therapies, while considering the patient's specific risk of complications related to both the disease and the treatment.^7^ In terms of adverse events, while vedolizumab is a good option, other alternatives, like ustekinumab, also have a promising efficacy‐safety ratio in CD patients.^4^, ^8^

Finally, understanding the mechanistic insights behind these observations from the systematic review and metanalysis is crucial and requires further biological characterization, particularly regarding the role of prior exposure to TNF antagonists which seems to reduce treatment persistence rate with vedolizumab.

## Data Availability

Data sharing not applicable to this article as no datasets were generated or analysed during the current study.
